# Is it worth including subtalar joint in ultrasound ankle assessment of patients with juvenile idiopathic arthritis?

**DOI:** 10.1186/1546-0096-12-S1-P36

**Published:** 2014-09-17

**Authors:** Stefano Lanni, Francesca Bovis, Francesca Magnaguagno, Angelo Ravelli, Stefania Viola, Annette Von Scheven-Gȇte, Nicola Ruperto, Alberto Martini, Clara Malattia

**Affiliations:** 1 Irccs Istituto Giannina Gaslini, Genova, Italy; 2 Universita' di Genova, Genova, Italy

## Introduction

The ankle is a complex anatomical structure owing to the multiple joint recesses and surrounding tendons. The involvement of subtalar joint (STJ) can be difficult to discern clinically from tibiotalar, tarsal or adjacent tendon disease. To date, only a few efforts have been centred around ultrasound (US)-detectable assessment of STJ in juvenile idiopathic arthritis (JIA).

## Objectives

1) To assess the frequency of US-detectable involvement of STJ, 2) To compare clinical versus US assessment in STJ detection, 3) To determine the most informative scanning approach to STJ.

## Methods

Fifty consecutive JIA patients, followed at the study center, with clinically-detected ankle involvement were enrolled. If both the ankles were involved, only the most affected side was selected for US. All clinical and US examinations were performed at the same day by experienced physicians and ultrasonographers, respectively, blinded to each other evaluations. US findings were collected using a lateral, medial and posterior STJ scanning approach. US synovitis was considered when both or either of joint effusion and synovial hypertrophy, with or without power Doppler signal, were visualized. Inter-observer reliability of US STJ involvement was tested using Cohen’s kappa coefficient in a subgroup of 24 patients. A control group of 10 healthy subjects was recruited.

## Results

None of the controls showed US STJ synovitis. US detected synovitis in 27 (54%) STJs of patients. Agreement between clinical and US assessment for presence and absence of STJ involvement was found in 17 (34%) and 16 (32%) ankles, respectively. In 10 (20%) STJs not considered to be clinically affected, synovitis was found on US. In 7 (14%) ankles labelled as having STJ involvement on clinical examination US was negative for STJ, but showed the involvement of different anatomical sites (midfoot, tibiotalar joint, tendons). Overall, the concordance between clinical and US evaluation was poor (k=0.32). The Cohen-kappa value for inter-observer reliability of STJ involvement on US was high (k= 0.92). All patients having US findings in the medial and/or posterior side of STJ presented also with US findings using the lateral scanning approach, but the reverse was not true.

## Conclusion

US is more sensitive than clinical evaluation in the assessment of STJ in ankles with active disease. The high frequency of its involvement may suggest to include the assessment of STJ in US scanning protocols. In this perspective, the lateral approach to the joint seems to be more appropriate for US evaluation of STJ involvement.

## Disclosure of interest

None declared.

